# Seahorses under a changing ocean: the impact of warming and acidification on the behaviour and physiology of a poor-swimming bony-armoured fish

**DOI:** 10.1093/conphys/cov009

**Published:** 2015-03-17

**Authors:** Filipa Faleiro, Miguel Baptista, Catarina Santos, Maria L Aurélio, Marta Pimentel, Maria Rita Pegado, José Ricardo Paula, Ricardo Calado, Tiago Repolho, Rui Rosa

**Affiliations:** af1 MARE - Marine and Environmental Sciences Centre, Faculdade de Ciências, Universidade de Lisboa, Laboratório Marítimo da Guia, Av. Nossa Senhora do Cabo 939, 2750-374 Cascais, Portugal; af2 Departamento de Biologia & CESAM, Universidade de Aveiro, Campus de Santiago, 3810-193 Aveiro, Portugal

**Keywords:** Acidification, behaviour, *Hippocampus guttulatus*, metabolism, ocean warming, seahorse

## Abstract

Seahorses are currently facing great challenges in the wild, including habitat degradation and overexploitation, and how they will endure additional stress from rapid climate change has yet to be determined. Unlike most fishes, the poor swimming skills of seahorses, along with the ecological and biological constraints of their unique lifestyle, place great weight on their physiological ability to cope with climate changes. In the present study, we evaluate the effects of ocean warming (+4°C) and acidification (ΔpH = −0.5 units) on the physiological and behavioural ecology of adult temperate seahorses, *Hippocampus guttulatus*. Adult seahorses were found to be relatively well prepared to face future changes in ocean temperature, but not the combined effect of warming and acidification. Seahorse metabolism increased normally with warming, and behavioural and feeding responses were not significantly affected. However, during hypercapnia the seahorses exhibited signs of lethargy (i.e. reduced activity levels) combined with a reduction of feeding and ventilation rates. Nonetheless, metabolic rates were not significantly affected. Future ocean changes, particularly ocean acidification, may further threaten seahorse conservation, turning these charismatic fishes into important flagship species for global climate change issues.

## Teaser

Many seahorse species are already threatened worldwide, and we do not know how they will endure an additional threat as climate change. Our results show that adult seahorses (*Hippocampus guttulatus*) seem to be relatively well prepared to face future changes in ocean temperature, but not the combined effect of warming and acidification.

## Introduction

Seahorses are charismatic fishes that catch people's attention with their unusual beauty and unique lifestyle. They are admired and sought all around the world, with millions of seahorses being traded every year for traditional medicine, aquarium and curio trade (Lourie *et al*., 2004). In recent decades, however, these fishes started to draw attention for the wrong reasons. Many seahorse species are currently threatened worldwide by intensive exploitation and overfishing, as well as by the widespread degradation of their natural habitats. Seahorses were included both in the IUCN Red List of Threatened Species and in the Appendix II of CITES, as well as in some regional and national lists of threatened species (Vincent *et al*., 2011).

The unique life-history features of seahorses make them particularly sensitive to overfishing and disturbance of their natural environment and, thus, an important and pressing focus for global conservation efforts (Vincent *et al*., 2011). Given the poor swimming skills and small home ranges of these fishes, migration to more suitable areas is somewhat restricted. Moreover, their camouflage defence strategy and sparse distribution associated with structurally complex habitats implies that not all habitats are equally suitable for them. Additionally, mate fidelity and the formation of pair bonds (which allows them to increase reproductive efficiency) means that when pair bonds are broken the seahorses may have trouble finding a new partner owing to their low population densities and sparse distribution; even when partners are replaced, their reproductive output may be affected. Reproductive rates of seahorses are already limited by the low fecundity, small brood size and lengthy parental care of these fish. Besides, male pregnancy implies that the survival of the young inside the brood pouch depends on the survival of the male (Foster and Vincent, 2004). Given the vulnerability of seahorses and the current concerns regarding their conservation, the question that arises is how will these already-threatened fish endure additional stress from rapid climate change?

The climate is changing at an unprecedented rate. Carbon dioxide (CO_2_) has greatly increased in the atmosphere, from pre-industrial levels of 280 ppm to present-day levels of 379 ppm, and is expected to continue rising up to 730–1020 ppm by the end of the century. The oceans act as carbon sinks, sequestering CO_2_ from the atmosphere. As a result, the continuous CO_2_ uptake by the oceans is projected to lead to a drop of 0.3–0.4 units in ocean pH by the year 2100. At the same time, the atmospheric temperature is rising due to increasing concentrations of greenhouse gases produced by human activities and is estimated to increase by a further 1.1–6.4°C by the end of the century. Consequently, the oceans are absorbing some of this heat from the atmosphere and becoming warmer (Meehl *et al*., 2007).

These expected environmental changes are predicted to have profound impacts on marine organisms and may trigger cascading effects on population, community and ecosystem dynamics (e.g. Pörtner *et al*., 2004, 2005; Fabry *et al*., 2008). Ocean warming will elicit thermal stress for a wide range of coastal marine organisms as their thermal limits are approached or even exceeded (Hofmann and Todgham, 2010). Beyond a certain thermal threshold, biological processes, such as metabolism, growth, feeding, reproduction and behaviour, may be negatively affected, compromising the overall fitness and survival of the species (e.g. Roessig *et al*., 2004; Hoegh-Guldberg *et al*., 2007; Byrne, 2011). Moreover, marine organisms at higher temperatures are likely to be more vulnerable to other environmental stressors, such as ocean acidification (e.g. Metzger *et al.*, 2007; Rosa and Seibel, 2008; Rosa *et al*., 2014). Increased CO_2_ levels may be responsible for reduced calcification rates and dissolution of calcareous structures in calcifying organisms (Hofmann *et al*., 2010) and may also affect the survival, growth, development, behaviour, digestion and respiratory physiology of marine species (e.g. Rosa and Seibel, 2008; Munday *et al*., 2009, 2011; Stumpp *et al*., 2012, 2013; Jutfelt *et al*., 2013).

In this study, we evaluate the effects of ocean warming (+4°C) and acidification (ΔpH = −0.5 unit) on the metabolism, ventilation rate, feed intake and behavioural patterns of adult temperate seahorses, *Hippocampus guttulatus*, in order to understand how these fish will respond to future changes in ocean temperature and chemistry. *Hippocampus guttulatus* is one of the two seahorse species that inhabit European waters and, like other seahorses, is currently facing great conservation concerns (Faleiro, 2011; Caldwell and Vincent, 2012).

## Materials and methods

### Specimen collection and stocking conditions

Adult seahorses, *H. guttulatus*, were collected using SCUBA diving equipment near Caldeira de Tróia, a shallow water habitat near the inlet of the Sado estuary (38°29′18.42″ N; 8°53′15.12″ W), on the west coast of Portugal.

Seahorses (ranging between 4 and 15 g) were acclimated for 1 month to different temperature and pH combinations. Temperature scenarios were chosen to reflect the following conditions: (i) the average annual temperature (18°C); (ii) the average temperature during summer heat waves (26°C); and (iii) the expected warming scenario (+4°C) for summer heat waves (30°C). All temperature scenarios were tested in conditions of normocapnia [0.04% CO_2_, partial pressure of CO_2_ (*p*CO_2_) = ∼400 µatm] and hypercapnia (0.14% CO_2_, *p*CO_2_ = ∼1400 µatm, ΔpH = −0.5 units), making a total of six experimental treatments.

Seahorses were kept in recirculating systems (three systems per treatment). In each system, one non-pregnant male and one female were individually incubated at a density of one seahorse per 38.3 litres, making a total of six seahorses per treatment. Systems were filled with filtered (1 µm) and ultraviolet (UV)-irradiated seawater (salinity 35) and illuminated with a photoperiod of 14 h light–10 h dark. Water quality was ensured using protein skimmers, bioballs and UV sterilizers. Concentrations of ammonia, nitrites and nitrates were monitored regularly and kept within recommended levels. Water changes of 20% were performed daily to maintain total alkalinity and dissolved inorganic carbon speciation due to bacterial activity. Seahorse husbandry conditions were defined based on previous studies with this species (see Faleiro *et al.*, 2008; Planas *et al.*, 2008). Plastic plants were provided as holdfasts. All fishes were wet-weighed and identified with coloured-bead necklaces loosely held around the neck with an elastic cotton string. Seahorses were fed *ad libitum* twice a day with frozen enriched adult *Artemia* and *Mysis*.

Water temperature and pH were monitored via a Profilux system connected to individual temperature and pH probes. Temperature was automatically upregulated by heaters and downregulated using chillers. The pH was adjusted automatically via solenoid valves; it was reduced by injection of a certified CO_2_ gas mixture or upregulated by aerating the tanks with CO_2_-filtered air. Additionally, seawater temperature and pH were manually monitored daily using a multiparameter portable meter, in order to cross-calibrate the temperature and pH probes and to adjust the set points of the systems as required. Seawater carbonate system speciation (see Supplementary Table S1) was calculated weekly from total alkalinity (see Sarazin *et al.*, 1999) and pH measurements. Bicarbonate and *p*CO_2_ values were calculated using the CO2SYS software, with dissociation constants from Mehrbach *et al.* (1973) as refitted by Dickson and Millero (1987).

After 1 month of acclimation, the metabolism, ventilation rates, feed intake and behavioural patterns of adult seahorses were analysed under the different climate change scenarios. At the end of the experiments, tags were removed and all seahorses were released into their original habitat without injuries.

### Metabolic rates, thermal sensitivity and ventilation rates

Oxygen consumption rates (OCRs) of adult seahorses were determined as an estimate of routine metabolic rates, after a starvation period of ∼24 h. Seahorses were individually placed within an intermittent flow-through respirometry set-up (Loligo Systems), filled with filtered (0.2 µm) and UV-irradiated seawater to avoid bacterial contamination. Holdfasts were provided for seahorse attachment. The water was pumped at a constant flow rate (average 140 ml min^−1^) through the respirometry chambers (550 ml vertical acrylic cylinders). During measurements of oxygen consumption, the flow was interrupted so that the system could operate like a closed respirometry set-up. Oxygen concentrations were recorded at the entrance and exit of each respirometer chamber with Clark-type O_2_ electrodes connected to a Strathkelvin Instruments 929 oxygen interface. Measurements were performed in temperature-controlled conditions using a Lauda water bath. Before and after each run, the experimental set-up was calibrated and checked for electrode drift and microbial oxygen consumption. Each experiment was 6 h long, comprising 2 h for acclimation and the following 4 h for oxygen measurement. As a result of weight dissimilarity between specimens, OCRs were later standardized to 10 g animals, assuming a scaling coefficient of −0.20 (see Clarke and Johnston, 1999).

Thermal sensitivity (*Q*_10_) was determined for the temperature intervals 18–26, 18–30 and 26–30°C, using the standard equation:

$$Q_{10}={\left(\frac{R_{2}}{R_{1}}\right)}^{\left[10/(T_{2}-T_{1})\right]},$$

where *R*_1_ and *R*_2_ represent the OCRs at temperatures “*T*_1_ and *T*_2_, respectively.

Ventilation rates were measured by counting the number of opercular beats per minute. This procedure was repeated five times per individual (over 5 consecutive days), immediately before feeding to exclude any potential bias caused by feeding on respiration.

### Feed intake and behavioural patterns

Seahorses were fed 100 frozen enriched adult *Artemia* twice a day. Feed intake was determined by collecting and counting the leftovers at the end of the day. Feed intake was later size corrected based on a daily feed intake of 5% of the body weight, in order to set aside size differences in this variable.

Seahorse behaviour was monitored through direct observation by blinded observers. In previous behavioural studies with this species (e.g. Faleiro *et al.*, 2008; Aurélio *et al.*, 2013), we were able to see that seahorses did not change normal behavioural patterns during passive and careful observation. Behavioural analysis was performed based on the ethogram described in Table 1 (adapted from Faleiro *et al.*, 2008). The time spent performing each activity was measured for each seahorse during 15 min per day and was later converted to a percentage of total time. This procedure was repeated five times per individual (over 5 consecutive days), comprising a total of 75 min of observation per seahorse and 450 min per treatment. Behavioural observations were made in the morning, 30 min after feeding.

**Table 1:** Ethogram of *Hippocampus guttulatus* activity patterns (adapted from Faleiro *et al.*, 2008)

Category	Behaviour	Description
Rest	Inactive	The seahorse remains resting, without performing any kind of movement, while attached or unattached to the holdfast
	Swinging	The seahorse remains attached to the holdfast, with slight movements of the head or body
Activity	Feeding	The seahorse tilts the body in search for food, points the snout towards the prey and swallows it
	Swimming	The seahorse swims, actively moving the dorsal and pectoral fins

### Statistical analysis

ANOVA was used to test for significant differences between sexes and seahorses from different systems of the same treatment. Given that no significant differences were found between them, all data from the same treatment were pooled and analysed together. The effects of the different temperature and pH scenarios on the OCRs, ventilation rates, feed intake and behavioural patterns of adult seahorses (*n* = 6) were then evaluated using two-way ANOVAs, followed by Tukey's *post hoc* tests. All statistical analyses were performed for a significance level of 0.05, using Statistica 10.0 software.

## Results

### Metabolic rates, thermal sensitivity and ventilation rates

**Figure 1:**
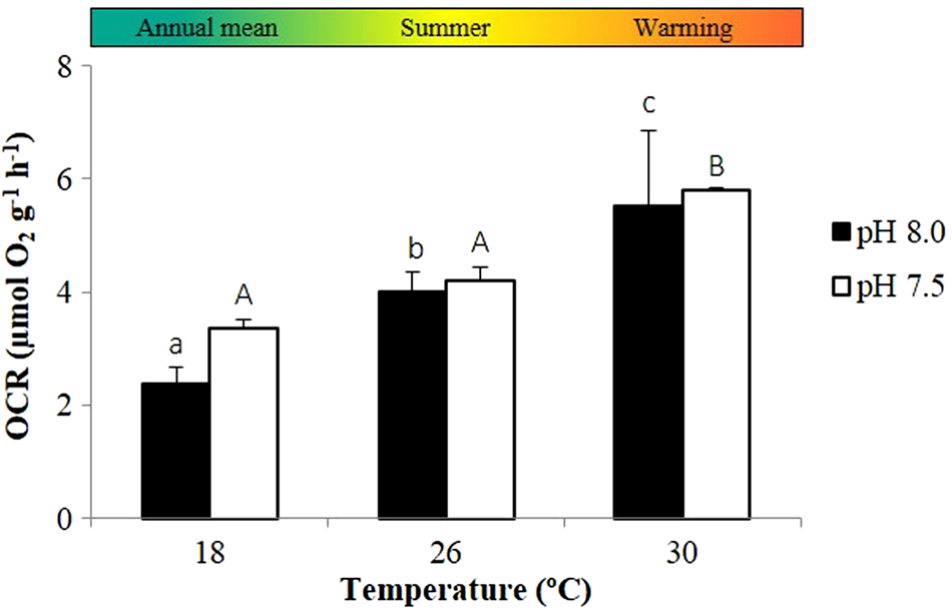
Impact of ocean warming and acidification on the oxygen consumption rates (OCRs) of adult seahorses, *H. guttulatus*, in resting conditions. Values are shown as means + SD (*n* = 6). Different lower case letters represent significant differences (*P* < 0.05) between temperatures at pH 8.0 and different upper case letters represent significant differences (*P* < 0.05) between temperatures at pH 7.5.

Ocean warming and acidification had a significant impact on the metabolism of *H. guttulatus* (Fig. 1 and Supplementary Table S2). The OCR of adult seahorses increased significantly with temperature in both normocapnic (*P* < 0.001) and hypercapnic conditions (*P* < 0.001). The future warming scenario promoted an increase in OCR from 3.97 ± 0.38 (at 26°C) to 5.47 ± 1.03 µmol O_2_ g^−1^ h^−1^ (at 30°C) during normocapnia and from 3.74 ± 0.19 (at 26°C) to 4.99 ± 0.00 µmol O_2_ g^−1^ h^−1^ (at 30°C) in acidified conditions. Thermal sensitivity varied between 1.25 and 2.22 depending on the climate scenario.

**Figure 2:**
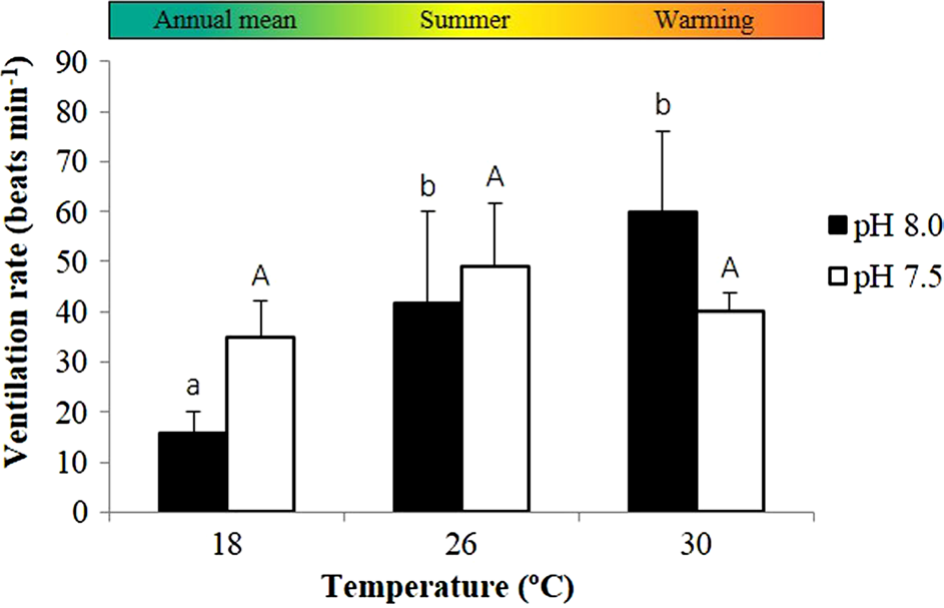
Impact of ocean warming and acidification on the ventilation rates of adult seahorses, *H. guttulatus*. Values are shown as means + SD (*n* = 6). Different lower case letters represent significant differences (*P* < 0.05) between temperatures at pH 8.0 and different upper case letters represent significant differences (*P* < 0.05) between temperatures at pH 7.5.

Ventilation rates of adult seahorses were also significantly affected by temperature (*P* < 0.001) and its interaction with pH (*P* = 0.001; Fig. 2 and Supplementary Table S2). During normocapnia, the ventilation rates followed the trend of metabolic rates, increasing with warming from 41.6 ± 18.3 (at 26°C) to 59.7 ± 16.2 beats min^−1^ (at 30°C). In the acidified scenario, however, the ventilation rates did not keep up with the increase in OCR, decreasing with warming from 49.2 ± 12.5 (at 26°C) to 40.2 ± 3.6 beats min^−1^ (at 30°C).

### Feed intake and behavioural patterns

**Figure 3:**
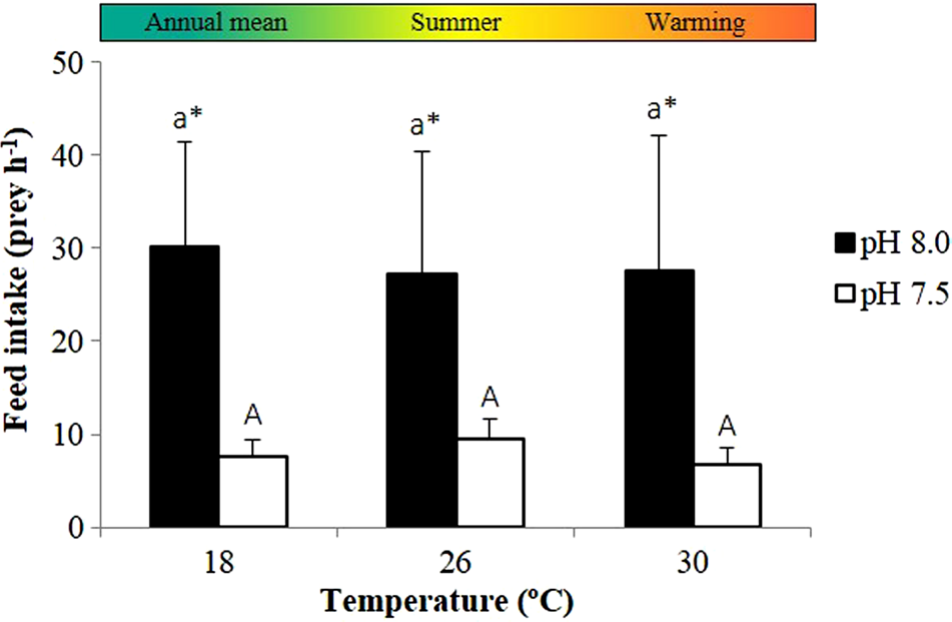
Impact of ocean warming and acidification on the feed intake of adult seahorses, *H. guttulatus*. Values are shown as means + SD (*n* = 6). Different letters and asterisks represent significant differences (*P* < 0.05) between temperature and pH conditions, respectively. Lower case letters are used for differences between temperatures at pH 8.0 and upper case letters for differences between temperatures at pH 7.5.

**Figure 4:**
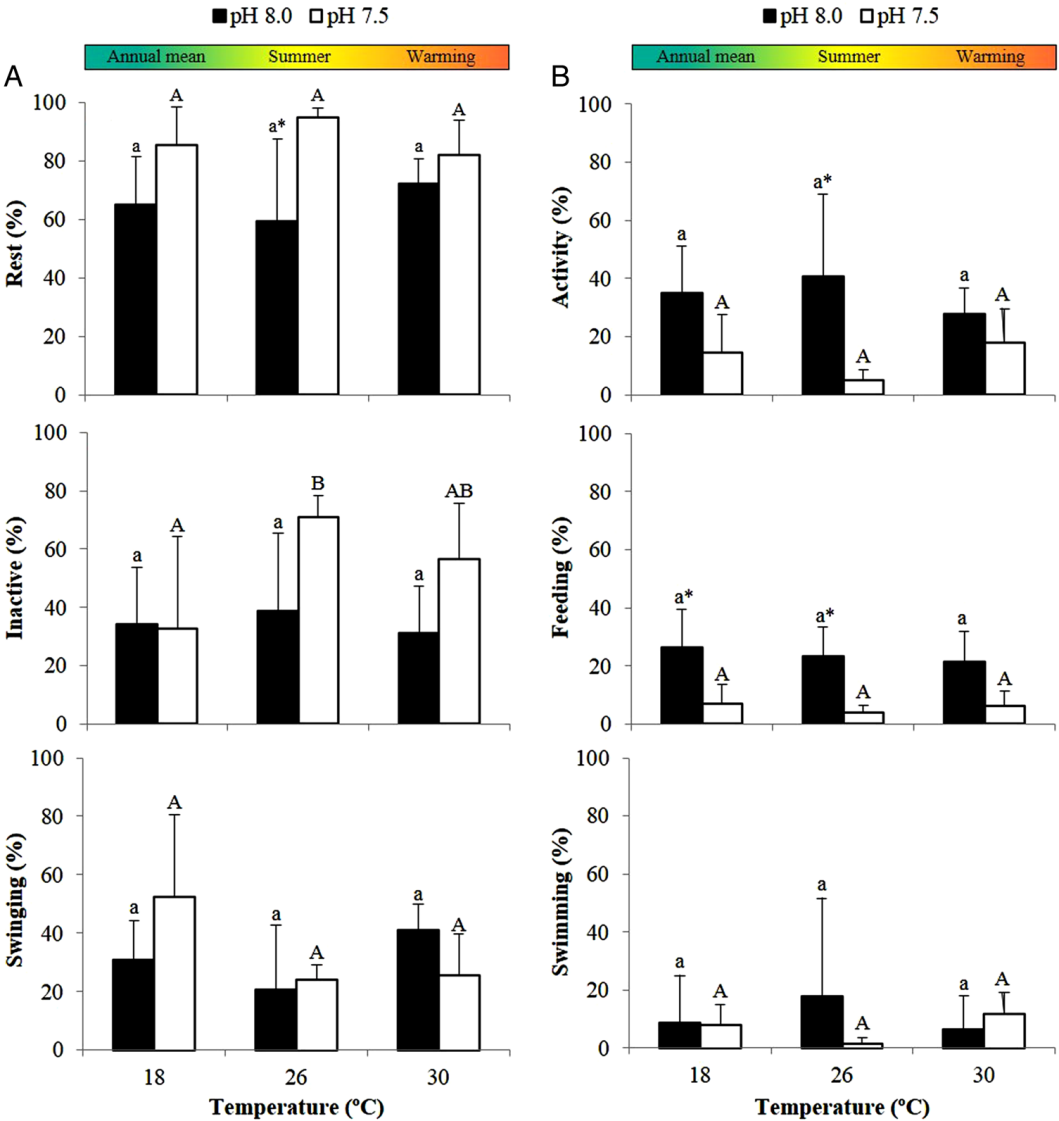
Impact of ocean warming and acidification on the behavioural patterns of adult seahorses, *H. guttulatus*. (**A**) Rest, which includes ‘inactive’ and ‘swinging’ behaviours. (**B**) Activity, which includes ‘feeding’ and ‘swimming’ behaviours. Values are shown as means + SD (*n* = 6). Different letters and asterisks represent significant differences (*P* < 0.05) between temperature and pH conditions, respectively. Lower case letters are used for differences between temperatures at pH 8.0 and upper case letters for differences between temperatures at pH 7.5.

The feed intake of adult seahorses did not increase with warming (*P* = 0.895), but was significantly affected by pH (*P* < 0.001; Fig. 3 and Supplementary Table S2). In acidified conditions, seahorse feeding was inhibited, which promoted a decrease in prey consumption from 27–30 to 7–10 prey h^−1^. Additionally, the seahorses in acidified conditions spent less time feeding than those in normocapnic conditions (*P* < 0.001; Fig. 4 and Supplementary Table S2).

In a similar way, seahorse behaviour remained almost unchanged with warming, but was influenced by pH (Fig. 4 and Supplementary Table S2). In hypercapnic conditions, seahorses spent less time in activity and more time resting (*P* < 0.001).

## Discussion

Phenotypic responses to climate change are already taking place in today's oceans; for instance, many animals are migrating polewards to more environmentally suitable areas (Perry *et al.*, 2005). Seahorses may not, however, benefit much from this strategy, because these poor swimmers are sparsely distributed in structurally complex habitats and rely on camouflage to survive. Moreover, migration is also expected to have profound impacts on their reproductive success, because it increases the chances of losing a mate. Seahorses establish faithful pair bonds that allow them to increase their reproductive efficiency and to avoid the risks of searching for a new mate. When pair bonds are broken, seahorses may have trouble finding a new partner due to reduced mate availability; even when partners are replaced, the reproductive output may be affected (Foster and Vincent, 2004). Thus, given these constraints, seahorses may have to rely more heavily than other fishes on their physiological ability to cope with predicted climate changes. In this study, we show that, even though adult seahorses may be relatively well prepared to face future changes in ocean temperature, they may not be able to cope with the combined effects of warming and acidification, which are predicted to act synergistically in the future ocean.

As expected for ectothermic animals, seahorse metabolism increased under the near-future warming scenario. Nonetheless, the increase in metabolic rates resulted in normal values of thermal sensitivity (*Q*_10_ values around 2), which suggests that adult seahorses were not under severe thermal stress. Indeed, seahorse behaviour remained reasonably unaffected by higher temperatures. The great thermal tolerance of *H. guttulatus* is not entirely unexpected, because these eurythermic animals are commonly found in shallow inshore waters, mainly in estuarial inlets and coastal lagoons (Lourie *et al.*, 2004), where they endure significant daily and seasonal temperature fluctuations. Their physiological ability to tolerate large temperature oscillations may thus give them a chance to face future increases in ocean temperature.

Although adult seahorses have shown great resilience to higher temperatures (see also Aurélio *et al.*, 2013), we cannot exclude potential negative impacts of long-term warming. In fact, in the present study there were some signs that seahorses may not be totally indifferent to higher temperatures. For instance, feeding rates did not follow the increase in metabolic rates with warming as expected. Whether similar feeding rates at higher temperatures will be enough to satisfy the greater energetic demands at these temperatures or if they will have a further negative impact on fish performance and welfare over a long-term perspective remains unclear. Long-term effects on vital biological features (such as survival, growth and reproduction) should be addressed further, in order to uncover potentially hidden impacts of ocean warming on adult seahorses.

In the present study, ocean acidification had a more pronounced impact on seahorses than warming. In conditions of lower pH, ventilation rates declined with rising temperature, feeding rates decreased considerably, and the activity levels were also reduced. This lethargic response may indicate that seahorses were struggling against a stress element. Indeed, as hypercapnia is known to enhance gill ventilation of fishes (Ishimatsu *et al.*, 2004), the decreased ventilation rates of seahorses in warming and acidified conditions may indicate acid–base imbalance. The consequences of this lethargic state during hypercapnia to long-term seahorse welfare remain unclear, but may ultimately affect growth rates and reproductive potential.

Fishes have evolved efficient ion-regulatory mechanisms that allow them to compensate pH rapidly and maintain their internal ionic environment through the exchange of ions across the gills (Pörtner *et al.*, 2004; Perry and Gilmour, 2006). Nonetheless, exposure to elevated CO_2_ has been shown to have detrimental effects on several aspects of fish life, including behaviour (e.g. Jutfelt *et al.*, 2013), growth and survival (e.g. Baumann *et al.*, 2011), skeletal and otolith development (e.g. Munday *et al.*, 2011), cardiovascular function (e.g. Ishimatsu *et al.*, 2004) and respiratory physiology (e.g. Munday *et al.*, 2009), even though most effects have been observed in fingerlings.

The combined effects of ocean warming and acidification had a negative impact on the behaviour and physiology of adult *H. guttulatus* seahorses. Moreover, their effects on early stages are expected to be much more severe, because newborns proved to be much more sensitive to warming than adult seahorses, evidencing signs of metabolic suppression when exposed to temperatures higher than 28°C (Aurélio *et al.*, 2013). Even though the effects of climate change on other seahorse species are still unknown, ocean warming and acidification may represent, in the near future, an extra threat to seahorse conservation. Seahorses already face several challenges in the wild (including habitat degradation and overexploitation), and climate change may increase their vulnerability further. Fortunately, given the time frame in which ocean warming and acidification are expected to occur, there is still an opportunity for adaptation.

## Supplementary material

Supplementary material is available at *Conservation Physiology* online.

## Funding

This work was supported by the Portuguese Foundation for Science and Technology (FCT) through a post-doctoral grant (SFRH/BPD/79038/2011) to F.F. and a project grant to R.R. (PTDC/AAG-GLO/3342/2012).

## Supplementary Material

Supplementary Data

## References

[COV009C1] Aurélio M Faleiro F Lopes VM Pires V Lopes AR Pimentel MS Repolho T Baptista M Narciso L Rosa R (2013) Physiological and behavioral responses of temperate seahorses (*Hippocampus guttulatus*) to environmental warming. Mar Biol 160: 2663–2670.

[COV009C2] Baumann H Talmage SC Gobler CJ (2011) Reduced early life growth and survival in a fish in direct response to increased carbon dioxide. Nat Clim Change 2: 38–41.

[COV009C3] Byrne M (2011) Impact of ocean warming and ocean acidification on marine invertebrate life history stages: vulnerabilities and potential for persistence in a changing ocean. Oceanogr Mar Biol 49: 1–42.

[COV009C4] Caldwell IR Vincent ACJ (2012) Revisiting two sympatric European seahorse species: apparent decline in the absence of exploitation. Aquat Conserv 22: 427–435.

[COV009C5] Clarke A Johnston NM (1999) Scaling of metabolic rate with body mass and temperature in teleost fish. J Anim Ecol 68: 893–905. 10.1111/j.1365-2656.2010.01672.x 20180875

[COV009C6] Dickson A Millero F (1987) A comparison of the equilibrium constants for the dissociation of carbonic acid in seawater media. Deep-Sea Res 34: 1733–1743.

[COV009C7] Fabry VJ Seibel BA Feely RA Orr JC (2008) Impacts of ocean acidification on marine fauna and ecosystem processes. ICES J Mar Sci 65: 414–432.

[COV009C8] Faleiro F (2011) A new home for the long-snouted seahorse, *Hippocampus guttulatus*: breeding in captivity to preserve in the wild. PhD thesis Faculty of Sciences, University of Lisbon, Lisbon.

[COV009C9] Faleiro F Narciso L Vicente L (2008) Seahorse behaviour and aquaculture: how to improve *Hippocampus guttulatus* husbandry and reproduction? Aquaculture 282: 33–40.

[COV009C10] Foster SJ Vincent ACJ (2004) Life history and ecology of seahorses: implications for conservation and management. J Fish Biol 65: 1–61.

[COV009C11] Hoegh-Guldberg O Mumby PJ Hooten AJ Steneck RS Greenfield P Gomez E Harvell CD Sale PF Edwards AJ Caldeira K (2007) Coral reefs under rapid climate change and ocean acidification. Science 318: 1737–1742. 18079392 10.1126/science.1152509

[COV009C12] Hofmann GE Todgham AE (2010) Living in the now: physiological mechanisms to tolerate a rapidly changing environment. Annu Rev Physiol 72: 127–145. 20148670 10.1146/annurev-physiol-021909-135900

[COV009C13] Hofmann GE Barry JP Edmunds PJ Gates RD Hutchins DA Klinger T Sewell MA (2010) The effect of ocean acidification on calcifying organisms in marine ecosystems: an organism-to-ecosystem perspective. Annu Rev Ecol Evol Syst 41: 127–147.

[COV009C14] Ishimatsu A Kikkawa T Hayashi M Lee KS Kita J (2004) Effects of CO_2_ on marine fish: larvae and adults. J Oceanogr 60: 731–741.

[COV009C15] Jutfelt F de Souza KB Vuylsteke A Sturve J (2013) Behavioural disturbances in a temperate fish exposed to sustained high-CO_2_ levels. PloS ONE 8: e65825. 23750274 10.1371/journal.pone.0065825 PMC3672104

[COV009C16] Lourie SA Foster SJ Cooper EW Vincent ACJ (2004) A Guide to the Identification of Seahorses. Project Seahorse and TRAFFIC North America, Washington, DC.

[COV009C17] Meehl GA Stocker TF Collins WD Friedlingstein P Gaye AT Gregory JM Kitoh A Knutti R Murphy JM Noda A (2007) Global climate projections. In Solomon S Qin D Manning M Chen Z Marquis M Averyt KB Tignor M Miller HL, eds, Climate Change 2007: the Physical Science Basis. Contribution of Working Group I to the Fourth Assessment Report of the Intergovernmental Panel on Climate Change. Cambridge University Press, Cambridge, UK, pp 747–845.

[COV009C18] Mehrbach C Culberson C Hawley J Pytkowicz R (1973) Measurement of the apparent dissociation constants of carbonic acid in seawater at atmospheric pressure. Limnol Oceanogr 18: 897–907.

[COV009C19] Metzger R Sartoris FJ Langenbuch M Pörtner HO (2007) Influence of elevated CO_2_ concentrations on thermal tolerance of the edible crab *Cancer pagurus*. J Therm Biol 32: 144–151.

[COV009C20] Munday PL Dixson DL Donelson JM Jones GP Pratchett MS Devitsina GV Døving KB (2009) Ocean acidification impairs olfactory discrimination and homing ability of a marine fish. Proc Natl Acad Sci USA 106: 1848–1852. 19188596 10.1073/pnas.0809996106 PMC2644126

[COV009C21] Munday PL Hernaman V Dixson DL Thorrold SR (2011) Effect of ocean acidification on otolith development in larvae of a tropical marine fish. Biogeosciences 8: 1631–1641.

[COV009C22] Perry SF Gilmour KM (2006) Acid–base balance and CO_2_ excretion in fish: unanswered questions and emerging models. Respir Physiol Neurobiol 154: 199–215. 16777496 10.1016/j.resp.2006.04.010

[COV009C23] Perry AL Low PJ Ellis JR Reynolds JD (2005) Climate change and distribution shifts in marine fishes. Science 308: 1912–1915. 15890845 10.1126/science.1111322

[COV009C24] Planas M Chamorro A Quintas P Vilar A (2008) Establishment and maintenance of threatened long-snouted seahorse, *Hippocampus guttulatus*, broodstock in captivity. Aquaculture 283: 19–28.

[COV009C25] Pörtner HO Langenbuch M Reipschlager A (2004) Biological impact of elevated ocean CO_2_ concentrations: lessons from animal physiology and earth history. J Oceanogr 60: 705–718.

[COV009C26] Pörtner HO Langenbuch M Michaelidis B (2005) Synergistic effects of temperature extremes, hypoxia, and increases in CO_2_ on marine animals: from earth history to global change. J Geophys Res-Oceans 110: C09S10.

[COV009C27] Roessig JM Woodley CM Cech JJ Hansen LJ (2004) Effects of global climate change on marine and estuarine fishes and fisheries. Rev Fish Biol Fish 14: 251–275.

[COV009C28] Rosa R Seibel BA (2008) Synergistic effects of climate-related variables suggest future physiological impairment in a top oceanic predator. Proc Natl Acad Sci USA 105: 20776–20780. 19075232 10.1073/pnas.0806886105 PMC2634909

[COV009C29] Rosa R Trübenbach K Pimentel MS Boavida-Portugal J Faleiro F Baptista M Calado R Pörtner HO Repolho T (2014) Differential impacts of ocean acidification and warming on winter and summer progeny of a coastal squid (*Loligo vulgaris*). J Exp Biol 217: 518–525. 24523499 10.1242/jeb.096081

[COV009C30] Sarazin G Michard G Prevot F (1999) A rapid and accurate spectroscopic method for alkalinity measurements in seawater samples. Water Res 33: 290–294.

[COV009C31] Stumpp M Hu MY Melzner F Gutowska MA Dorey N Himmerkus N Holtmann WC Dupont ST Thorndyke MC Bleich M (2012) Acidified seawater impacts sea urchin larvae pH regulatory systems relevant for calcification. Proc Natl Acad Sci USA 109: 18192–18197. 23077257 10.1073/pnas.1209174109 PMC3497771

[COV009C32] Stumpp M Hu M Casties I Saborowski R Bleich M Melzner F Dupont S (2013) Digestion in sea urchin larvae impaired under ocean acidification. Nat Clim Change 3: 1044–1049.

[COV009C33] Vincent ACJ Foster SJ Koldewey HJ (2011) Conservation and management of seahorses and other Syngnathidae. J Fish Biol 78: 1681–1724. 21651523 10.1111/j.1095-8649.2011.03003.x

