# Supplementary material for: Seahorses under a changing ocean: the impact of warming and acidification on the behaviour and physiology of a poor-swimming bony-armoured fish
Source: Conserv Physiol. 2015 Mar 17;3(1):cov009. doi: 10.1093/conphys/cov009 (PMC4778478; doi:10.1093/conphys/cov009)
Supplement: Supplementary Data [file cov009supp.zip › cov009supp.docx]

**SUPPLEMENTARY MATERIAL**

**Supplementary Table S1.** Seawater carbonate chemistry data for the different climate change scenarios. Total carbon (C_T_), carbon dioxide partial pressure (*p*CO_2_), bicarbonate concentration (HCO_3_^-^) and aragonite saturation state of seawater (Ω_arag_) were calculated with CO2SYS using temperature, pH, salinity and total alkalinity (A_T_). Values represent means ± SD.

| **Temperature**  **(ºC)** | **pH**  **(total scale)** | **Salinity** | **A_T_**  **(µmol ,kg^-1^ SW)** | **C_T_**  **(µmol kg^-1^ SW)** | ***p*CO_2_**  **(µatm)** | **HCO_3_^-^**  **(µmol kg^-1^ SW)** | **Ω_arag_** |
| --- | --- | --- | --- | --- | --- | --- | --- |
| 18.1 ± 0.4 | 8.01 ± 0.04 | 35.1 ± 0.5 | 1956.0 ± 34.1 | 1749.1 ± 31.8 | 367.2 ± 66.7 | 1596.0 ± 29.0 | 2.17 ± 0.03 |
| 18.4 ± 0.3 | 7.54 ± 0.03 | 35.3 ± 0.7 | 2171.0 ± 34.1 | 2124.0 ± 64.1 | 1342.0 ± 40.5 | 2017.0 ± 60.8 | 0.95 ± 0.02 |
| 25.9 ± 0.7 | 8.02 ± 0.05 | 35.4 ± 0.9 | 2050.2 ± 26.6 | 1772.7 ± 24.6 | 371.9 ± 51.9 | 1572.9 ± 40.4 | 3.01 ± 0.34 |
| 26.2 ± 0.5 | 7.52 ± 0.04 | 34.9 ± 0.8 | 2024.4 ± 16.2 | 1956.9 ± 11.6 | 1383.8 ± 113.6 | 1848.9 ± 11.8 | 1.12 ± 0.08 |
| 29.9 ± 0.2 | 8.00 ± 0.07 | 35.7 ± 1.1 | 2046.9 ± 27.1 | 1740.8 ± 72.0 | 382.2 ± 87.5 | 1523.9 ± 97.2 | 3.35 ± 0.43 |
| 30.1 ± 0.5 | 7.49 ± 0.03 | 35.8 ± 0.8 | 2030.3 ± 36.6 | 1952.5 ± 11.1 | 1481.1 ± 107.7 | 1839.0 ± 13.5 | 1.24 ± 0.20 |

**Supplementary Table S2.** Results of two-way ANOVA evaluating the effects of ocean warming and acidification on oxygen consumption rates, ventilation rates, feed intake and behavioural patterns of adult seahorses, *Hippocampus guttulatus*.

| **Measure** | **Factor** | **df** | **F** | **p** |
| --- | --- | --- | --- | --- |
| Oxygen consumption rates | Temperature (*T*) | 2 | 83.61 | 0.000 |
|  | pH | 1 | 00.04 | 0.839 |
|  | *T* x pH | 2 | 06.15 | 0.006 |
|  | Error | 30 |  |  |
| Ventilation rates | Temperature (*T*) | 2 | 14.73 | 0.000 |
|  | pH | 1 | 00.38 | 0.544 |
|  | *T* x pH | 2 | 08.46 | 0.001 |
|  | Error | 30 |  |  |
| Feed intake | Temperature (*T*) | 2 | 00.11 | 0.895 |
|  | pH | 1 | 42.41 | 0.000 |
|  | *T* x pH | 2 | 00.20 | 0.817 |
|  | Error | 30 |  |  |
| % Rest (% Activity) | Temperature (*T*) | 2 | 00.06 | 0.946 |
|  | pH | 1 | 17.77 | 0.000 |
|  | *T* x pH | 2 | 02.04 | 0.148 |
|  | Error | 30 |  |  |
| % Inactive | Temperature (*T*) | 2 | 02.94 | 0.068 |
|  | pH | 1 | 06.80 | 0.014 |
|  | *T* x pH | 2 | 02.07 | 0.144 |
|  | Error | 30 |  |  |
| % Swinging | Temperature (*T*) | 2 | 03.87 | 0.032 |
|  | pH | 1 | 00.32 | 0.577 |
|  | *T* x pH | 2 | 03.49 | 0.043 |
|  | Error | 30 |  |  |
| % Feeding | Temperature (*T*) | 2 | 00.46 | 0.637 |
|  | pH | 1 | 37.04 | 0.000 |
|  | *T* x pH | 2 | 00.19 | 0.825 |
|  | Error | 30 |  |  |
| % Swimming | Temperature (*T*) | 2 | 00.02 | 0.980 |
|  | pH | 1 | 00.51 | 0.480 |
|  | *T* x pH | 2 | 01.35 | 0.273 |
|  | Error | 30 |  |  |
